# Looking at Complicating Non-Biological Issues in Women with HIV

**DOI:** 10.4103/0974-777X.59246

**Published:** 2010

**Authors:** Chaturaka Rodrigo, Senaka Rajapakse

**Affiliations:** *University Medical Unit, National Hospital of Sri Lanka, Sri Lanka*; 1 *Department of Clinical Medicine, Faculty of Medicine, University of Colombo, Sri Lanka*

**Keywords:** HIV/AIDS, Women, Gender

## Abstract

**Introduction:**

The increasing number of women acquiring Human Immunodeficiency Virus (HIV) has resulted in a ‘feminization’ of the epidemic. In this article we are reviewing whether females are disadvantaged in the epidemic, due to factors independent of the biological differences in sexes.

**Materials and Methods:**

We searched MEDLINE and EMBASE for articles with key words ‘Women’, ‘Gender,’ and ‘HIV’ in any field. The search was restricted to articles published in English within the last 10 years (1999-2009). Data were coded independently by two reviewers from 94 selected sources. The coded data were categorized under five commonly encountered concepts; violence, poverty, gender norms, prevention-/treatment-related issues, and Highly Active Anti-Retroviral Treatment (HAART).

**Results:**

The link between inter-partner violence (IPV) and HIV risk for women is observed by many authors. In assessing the link between poverty and HIV, indicators such as food insufficiency and income inequality may be better indicators compared to wealth itself. Although women are disadvantaged with male-dominated gender norms, evidence suggests that the traditional norms are changing in many societies. A positive association between living in urban communities, education, and better HIV knowledge has been observed in females, although it is not always synonymous with reduced risk behavior.

**Conclusions:**

Women are still disadvantaged in many HIV-related issues such as poverty, violence, and gender norms. At least in Africa, there is evidence of a positive change in spheres of education and gender norms. However, the situation in Asia is largely unexplored.

## INTRODUCTION

The latest estimates of the HIV (Human Immunodeficiency Virus) epidemic suggest that 33 million people live with it worldwide. Fifty percent of them are women.[1] However, in 1985, only 35% of the infected were women.[2] The main mode of transmission has shifted from male homosexual contact to heterosexual contact over the last 20 years. More women getting infected via heterosexual intercourse has resulted in ‘feminization of HIV/AIDS’.[2] The percentage of infected women varies in different regions, but the numbers are increasing (Sub-Saharan Africa = 60%, Caribbean = 45-50%, Asia and Latin America = 30-40%, Eastern Europe and Central Asia = 30%).[1] This changing face of the epidemic calls for a careful review of gender-related differences of HIV.

An analysis of sexual behavior in 59 countries has shown that in many developing countries the age of sexual initiation has risen for women. Still, due to the late age in marriage, the prevalence of premarital sex has increased.[3] This and other trends in sexual behavior are dependent on local factors such as poverty, culture, gender norms, media influence, and so on. Transmission of HIV and other sexually transmitted diseases (STDs) are also influenced by these issues, which may affect men and women differently. Identifying these factors and their gendered effects will have major implications in prevention strategies.

During the synthesis of our review, it seemed logical to broadly categorize the gender-related variables as biological and nonbiological. The clinical manifestations, differences in pathogenesis, pregnancy-related issues, response to HAART, and side effects are some biological issues. As the scope of the topic is vast, we restricted our review to examine only the nonbiological factors, with gender implications, and how they relate to women at risk and women living with HIV/AIDS.

## MATERIALS AND METHODS

We searched the electronic databases; MEDLINE and EMBASE with the keywords ‘Women’ ‘Gender,’ and ‘HIV’ in any f ield, using the software Endnote X1.01, to the filter articles. The search was limited to articles published in English over the last 10 years (1999-2009). Bibliographies of cited literature were also searched. Relevant publications and epidemiological data were also downloaded from websites of international agencies such as the World Bank, United Nations Joint Program on HIV/AIDS (UNAIDS), and World Health Organization (WHO). All abstracts were read independently by the two authors, and relevant articles were identified for review of the full articles.

Sources were screened for relevance to the topic, originality, a well-described methodology, rigor of statistical analysis, and an adequate sample size, where relevant. Sources were excluded where sample size was inadequate (except in qualitative studies), methodology was not well-described, statistical analysis was unclear, and where the full article was unavailable. Ninety four sources from a selected 115 (81.7%) were reviewed. Coding was done by two reviewers independently blinded to each other. Codes were grouped into concepts commonly encountered (e.g., gender norms, violence, poverty, treatment- and prevention-related issues) during the search, under the broader category of nonbiological factors. Data sources included reviews published in core clinical journals, cohort studies, qualitative studies, interventional studies, case-control studies, cross-sectional analysis, and epidemiological data. The inter-reviewer agreement for data included in the final synthesis was 100%. A summary of cited literature is shown in Tables 1–3.

**Table 1:** HIV and women; a look into the nonbiological factors complicating the picture. A brief summary of studies quoted in text (in order of citation)

Author	Year published	Sample size	Study design	Conclusions
Andersson *et al.*	2007	20,639	Cross-sectional survey in eight Southern African countries	Eighteen percent of women reported partner physical violence within one year.
Garcia-Moreno *et al.*	2006	24,097	Population based survey, 2000-2003	The reported lifetime prevalence of physical or sexual partner violence or both ranged from 15-71%
*Sa et al.*	2008	1418	Population-based survey of women	Significant risk of HIV* was associated with a partner older than 10 years, if he made a low financial contribution to children's needs or if she experienced coerced sex before 18 years of age
Fonck *et al.*	2005	520	Survey of women attending STI clinics in Nairobi, Kenya	Prevalence of lifetime physical violence was 26%, mainly by an intimate partner (74%)
Dunkle *et al.*	2004	1366	Cross-sectional study of women presenting at antenatal clinics	Intimate partner violence and high levels of male control in a woman's current relationship were associated with HIV* seropositivity
Jewkes *et al.*	2006	1295	Cluster randomized controlled trial in South African women aged 15-26 with HIV* behavioral intervention	Inter-partner violence was strongly associated with most of the identified HIV* risk factors (past year partner numbers, time of last sex, and partner's education).
Pettifor *et al.*	2004	11,904	Household survey of men and women aged 15-24 in South Africa	Women experiencing forced sex were 5.77 times more likely to use condoms inconsistently. Inconsistent condom use was, in turn, significantly associated with HIV* infection
Langen TT	2005	2658	Women aged 18-49 years in a cross-sectional survey in Kwa Zulu Natal Province of South Africa and Botswana	Women with partners 10 or more years older than them, abused women, and those economically dependent on their partners are less likely to suggest condom use to their partners
Pronyk *et al.*	2006	6576 (In all three arms)	Cluster randomized intervention trial	A combined microfinance and training intervention can lead to reductions in levels of intimate-partner violence in program participants
Voisin DR	2005	409	Cross-sectional survey of adolescents in the United States of America	Adolescents exposed to either childhood sexual abuse or family or community violence were almost three and four times more likely than peers not exposed to such violence to report a higher number of HIV*-related risk behaviors
Raj *et al.*	2004	170	Community-based survey on Hispanic women in the United States of America	Abused women were significantly more likely than those not abused in the past three months to report high STD†^/^HIV* risk, gender-based risk, including sexual control by male partners and male partner risk including male infidelity
Martin *et al.*	1999	6632	Cross-sectional community-based survey of men in Uttar Pradesh, India	Men having extramarital sex and STD† symptoms were more likely to abuse their wives
Rowley *et al.*	2008	1743	Cluster survey in Lugufu refugee camp, Tanzania	Factors that may increase the risk of HIV* infections among refugees compared to the population in the surrounding villages include young age of sexual initiation among males, high-risk sex partners in the 15-24 year age group, limited access to income, and the vulnerability of refugee women totransactional sex
Silverman *et al.*	2007	287	Retrospective analytical study of sex-trafficked Nepali women	Repatriated Nepalese sex-trafficked girls and women were found to have a high prevalence of HIV* infection, with increased risk among those trafficked prior to the age of 15 years
Sarkar *et al.*	2008	580	Community-based cross-sectional survey of sex workers in Eastern India	The seroprevalence of HIV* was strikingly higher among Nepalese (43%) than among Bangladeshis (7%) and Indians (9%).
Lau *et al.*	2009	728	Two point cross-sectional analysis of clients of female sex workers in China following an intervention safe sex	Condom use and service utilization behaviors changed in the study population over a time period, when an enhanced intervention program was implemented
Subramanian *et al.*	2008	4821	Cross-sectional survey of clients of female sex workers in Andhra Pradesh, India	The prevalence of HIV* ranged from 2.0 to 10.9%, syphilis ranged from 3.1 to 10.1%; gonorrhea and Chlamydia ranged between 0 and 4.5%
Patterson *et al.*	2009	400	Cross-sectional survey of clients of female sex workers in Mexico	Although sexually transmitted infection prevalence was lower than among female sex workers, HIV* prevalence was comparable, suggesting the need for interventions among clients
Cohen *et al.*	2000	1645	Survey of women with HIV* or at risk of HIV*	A continuum of risk, with early childhood abuse leading to later domestic violence, may increase the risk of behaviors leading to HIV* infection
McDonnell *et al.*	2003	611	Quantitative and qualitative assessment of HIV* positive and demographically similar HIV* negative women	Significant differences were found between HIV*-positive women and HIV*-negative women in the pattern of abuse experienced as children, the frequency of abuse as an adult, and the involvement of women's drinking before or during a violent episode
McDonnell *et al.*	2005	445 (188 HIV* + 257 HIV* -)	Quantitative and qualitative assessment	Living with HIV* or having experienced inter-partner violence in the past year was significantly associated with poorer levels of the health-related quality of life
Gielen *et al.*	2000	310	Quantitative and qualitative assessment of HIV* positive women in the United States of America	Although 4% reported physical abuse following a disclosure event, 45% reported experiencing emotional, physical, or sexual abuse some time after their diagnosis
Maman *et al.*	2002	245	Follow-up study of HIV* positive and negative women for three months after HIV* testing	The odds of reporting at least one violent event was significantly higher among HIV*-positive women than among HIV-negative women (OR^‡^-2.63)

* HIV-Human immunodeficiency virus

† STD-Sexually transmitted diseases, OR-Odds ratio

**Table 2:** HIV and women; a look into the nonbiological factors complicating the picture. A brief summary of studies quoted in text (in order of citation, continued from Table 1)

Author	Year published	Sample size	Study design	Conclusions
Gaillard *et al.*	2002	290	Intervention study of HIV*-positive women in Kenya	Despite careful counseling, 10% subsequently experienced violence or disruption of their relationship
Talbott *et al.*	2007		Analytical study of UNAIDS§ data for 77 countries	Significant correlation exists between a countries' HIV* prevalence and income inequality, but not with wealth
Adimora *et al.*	2006	493 cases, 1063 controls	Population-based case-control study of black men and women, aged between 18 and 61 years in North Carolina	Poverty may be an underlying determinant of high risk behaviors and a contributor to infection risk even in people who do not have high-risk behaviors
Dunkle *et al.*	2004	1366	Cross-sectional study of women seeking antenatal care in Soweto, South Africa	Transactional sex may place women at an increased risk of HIV*, and is associated with gender-based violence, substance use, and socio-economic disadvantage
Hargreaves *et al.*	2002	1000 men, 1000 women	Cross-sectional population survey of adults from an urban population, Kenya	Risk profiles suggested that men and women of lower socioeconomic status may be at a greater risk of newly acquired HIV* infection
Gavin *et al.*	2006	1807	Cross-sectional analysis combined with HIV* testing for females aged between 15 and 19 years in Zimbabwe	Adolescent females in Zimbabwe who are married, not attending school and/or are unemployed, are at a higher risk of HIV* infection
Weiser *et al.*	2007	2051	Cross-sectional population-based study of adults in Botswana and Swaziland	Food insufficiency was associated with inconsistent condom use with a non-primary partner, sex exchange, intergenerational sexual relationships, and lack of control in sexual relationships.
Oyefara *et al.*	2005	320	Cross-sectional survey and qualitative interviews of female sex workers in Lagos, Nigeria	There is a significant relationship between poverty, food insecurity, and use of condoms by sex workers at a *P* < 0.01
Seib *et al.*	2009	247 sex workers, 185 clients	Cross-sectional analysis of sex workers and clients in Australia	There was little variation in self-reported lifetime STD† prevalence of licensed brothel, private and illegal (predominantly street-based) sex workers, although licensed brothel workers have been less likely to report ever being diagnosed with gonorrhea or pubic lice in the past (*P* = 0.035 and 0.004, respectively).
Mercer *et al.*	2007	1175 women, 703 men	Cross-sectional survey of rural married adults in Bangladesh	The proportions of men who reported sex with a female sex worker or with another male, while living away, were double the proportions reporting they had done so before living away or among men who had not lived away (*P* < 0.05).
Go *et al.*	2003	84	In-depth interviews and focus group discussions with adult men and women from slum communities of Chennai, India	Given the choice between the immediate threat of violence and the relatively hypothetical risk of HIV*, women often resign themselves to sexual demands and indiscretions that may increase their risk of HIV* acquisition
Luginaah *et al.*	2005	Two focus groups	In-depth interviews and focus group discussions with widows and community leaders in Nyanza, Kenya	Wife inheritance, emerged as an outstanding issue forthe widows in the context of HIV* transmission
Voisin *et al.*	2006	280	Survey among detained female adolescents in the United States of America	Greater substance use, stronger risk-taking attitudes, lower perceived parental monitoring, and familial support, gender roles supporting male dominance, risky peer norms, and lower student-teacher connectedness, were independently associated with increased STD† risk behaviors
Harrison *et al.*	2006	101 male, 199 female adolescents	Survey on adolescents aged between 18 and 24 years in South Africa	For men, more frequent condom use was not only associated with higher levels of partner attachment, but also with stronger approval of relationship violence and dominant behavior. In contrast, for women, more frequent condom use was correlated with a lower endorsement of relationship violence
Kaufman *et al.*	2008	309 males	Survey of men attending STD† services clinic in Cape Town, South Africa	A negative attitude toward women was significantly positively associated with a high level of HIV* risk behavior, and that endorsement of traditional male roles was negatively associated with HIV* risk behavior
Hebling *et al.*	2004	Six focus groups	Qualitative study with focus group discussions associated with a prospective interventional study	Although women had information about HIV*, they did not use preventive measures in steady relationships because they did not wield their decision-making power. Gender inequality and fidelity were two significant issues related to the increase in HIV* among women
Kerrigan *et al.*	2007	50	In-depth interviews with African American adolescents in Boston, United States of America	Stronger adherence to female gender ideologies related to emotional strength and caretaking may be linked to a heightened desire for male intimacy and tolerance of male sexual risk behavior, with increased risk of HIV* transmission
Stephenson *et al.*	2009	11,410	Analysisof demographic health survey data for men and women aged between 15 and 24 years, in Ghana, Burkina Faso, and Zambia	For young women, residence in communities with demographic and behavioral patterns that are indicative of greater opportunities are associated with increased knowledge of HIV
Smith *et al.*	2004	863	Cross-sectional analysis combined with in-depth interviews of adolescents and unmarried young adults	Assessments of current and potential partners, choices about whether or not to have sex, and decisions about whether or not to use condoms are influenced by shared cultural values regarding the importance of parenthood. These are gender-specific and put men and women in different negotiating positions with regard to sex and contraception
Jarama *et al.*	2007	51	Qualitative study among African American women	Despite having risk behaviors, all women in the sample perceived their risk of infection to be nonexistent
Prata *et al.*	2005	1995	Knowledge, attitudes and practices survey in Luanda, Angola among youth aged between 15 and 24 years	Urban residence, higher education, being in school and not equating condom use with lack of trust were important predictors of use at last intercourse in regular and casual relationships, whereas, access to condoms was the most important factor in spousal relationships
de Walque *et al.*	2005	Round 1 Females: 1627 Males: 1388 Round 2 Females: 2271 Males: 1876	Cohort study in Uganda with follow-up for 11 years	In 1989-1990, higher educational attainment was associated with higher risk of HIV*-1 infection, especially among males, but once odds ratios were adjusted for age, no significant relation remained. In 1999-2000, there was, for females aged 18 – 29 years, a significant relationship between higher educational attainment and lower HIV* prevalence. (*P* for trend 0.01)
Barden-O'Fallon *et al.*	2004	1601	Community-based survey in rural Malawi	Knowledge of HIV does not necessarily translate into perceived risk. In addition, there appears to be a gender difference where men assess risk in a relationship to their own behavior, while women do so in relation to their partner's behavior

* HIV-Human immunodeficiency virus

† STD- Sexually transmitted diseases

§ UNAIDS- United Nations Joint Programme on HIV/AIDS

**Table 3:** HIV and women; a look in to the nonbiological factors complicating the picture. A brief summary of studies quoted in text (in order of citation, continued from Table 2)

Author	Year published	Sample size	Study design	Conclusions
Mansoor *et al.*	2008	1054	Survey among First year students in Afghan Universities	Females were statistically more knowledgeable than males, and high-risk behaviors were significantly more prevalent among males; *P* = 0.01 and *P* = 0.001, respectively
Li *et al.*	2004	1081	Survey on Chinese College students	Males were more knowledgeable than females overall (*P* < 0.0001)
McManus *et al.*	2008	251	Cross-sectional survey on girls in secondary schools in New Delhi, India	About 30% of respondents considered HIV*/AIDS‖ could be cured, 49% felt that condoms should not be available to youth, 41% were confused about whether the contraceptive pill could protect against HIV* infection and 32%thought it should only betaken by married women
DiClemente *et al.*	2004	522	Randomized controlled trial of African American girls aged between 14 and 18 years. The intervention group underwent a comprehensive education programme	Over the 12-month follow-up, adolescents in the intervention were more likely to use a condom at the last intercourse, less likely to have a new vaginal sex partner in the prior 30 days, and more likely to apply condoms to sex partners and they had better condom application skills, a higher percentage of condom-protected sexacts, and fewer unprotected vaginal sex acts
Hirsl-Hećej *et al.*	2006	2070 in first round, 1972 in second round	Two-wave cross-sectional analysis of Croatian adolescents in a four-year interval	Statistically significant increases in knowledge of HIV* and condom use. Both changes were more substantial among female students
Chhabra *et al.*	2008	1846	Interventional study to assess school-based educational program in Mumbai, India	Both boys and girls significantly improved their knowledge, attitudes, and beliefs regarding HIV*/AIDS‖ and in their confidence level in dealing with risky behavior. However girls showed significantly better results on comparison
Di *Noia *et al.**	2007	204	Interventional study to assess an educational program on HIV* prevention	Improved HIV* knowledge and understanding of avoidance of risk behavior compared to controls
Ndiaye *et al.*	2009	318	Cross-sectional analysis of HIV*-positive males and females in Mali and Burkina Faso on the sero status disclosure	Cohabiting with the partner was strongly associated with disclosure in both men and women. In men only, older age, literacy, and having good communication with the treating doctor were significantly associated with disclosure. Among women, having children and high self-reported importance of religion was associated with disclosure
Arrington-Sanders *et al.*	2008	369	Prevalence of HIV* testing among sexually active African Americans	The adjusted odds of HIV* testing was 2.7 times higher for female adolescents than males
Jarrin *et al.*	2008	6923	Retrospective analysis of 23 HIV* seroconverter cohort studies from Europe, Australia, and Canada	From 1997 onward, women had a lower risk of AIDS‖ (adjusted cumulative relative risk = 0.76) and death (adjusted hazard ratio = 0.68) than men
Moore *et al.*	2002	497 males, 146 females	Prospective cohort study after initiation of HAART^¶^	A possible benefit in women compared to men, in the rate of outcomes after HAART^¶^
Chen *et al.*	2008	2838	Retrospective cohort study	Significantly higher survival rates among females than males in WHO** stages 1, 2, and 3 (both *P* < 0.0001) and borderline in stage 4 (*P* = 0.076)
Chandra *et al.*	2009	log	Cross-sectional analysis of quality of life using WHO** quality of life instrument for HIV* in men and women, living with HIV*, in South India	Men reported significantly higher quality of life in the following facets — positive feeling, sexual activity, financial resources, and transport, while women reported significantly higher quality of life on the forgiveness and blame facet
Braitstein *et al.*	2008	33,164	A comparison of UN AIDS§ data on the gender distribution of HIV* infection with the proportions of women and men receiving HAART^¶^ in the ART-LINC Collaboration (Clinic network in Asia, Africa, and Latin America)	Women in resource-constrained settings are not necessarily disadvantaged in their access to HAART^¶^
Cook *et al.*	2007	1710	Prospective cohort study on HIV* positive women in the United States of America	There were interactive effects of drug use and depressive symptoms on reduced likelihood of HAART^¶^
Mathews *et al.*	2002	235	Prospective cohort study on predictors of adherence and outcome after initiation of HAAR^¶^	Males were more adherent than females (*P* < 0.05) to HAART^¶^ regimes
Turner *et al.*	2003	5073	Prospective cohort study on HIV* positive individuals on HAAR^¶^ in the United States of America	Women were less adherent than men (18 vs. 25%, respectively, *P* < 0.001) and more likely to be diagnosed with depression (34 vs. 29%)
Berg *et al.*	2004	113	Prospective cohort study on HIV* positive current or former opioid users	Median adherence among women was 27% lower than among men (46 vs. 73%; *P* < 0.05)
Applebaum *et al.*	2009	67	Analysis of adherence to treatment regimes in HIV*-positive individuals	No significant difference in adherence was observed between men and women
Solomon *et al.*	2008	356	Cross-sectional analysis of HIV*-positive people in South India	Men and women reported similar scores in physical well-being, satisfaction with healthcare, and relationship with partner from the period prior to care, at enrollment, and at 6 months. Women scored significantly lower than men in psychosocial well-being from the period prior to care, at enrollment, and at six months (*P* < 0.05)
Shor-Posner *et al.*	2000	75	Prospective cohort study of intravenous drug using HIV* positive individuals	Significant gender differences were observed in activity assessment. Independent of disease status, women had significantly lower activity scores (*P* = 0.0038)
Strebel *et al.*	2006		Qualitative study with in-depth interviews and focus group discussions	The familiar issues of stigma, denial, and disclosure emerged in both interviews and focus groups. Use of condoms was difficult. HIV* positive women suffered differently from stigma compared to men
Mantell *et al.*	2009	10 semi-structured focus group discussions	Qualitative study among tertiary institution students in South Africa	Significant changes in the post-apartheid era, particularly for emerging gender norms that promote women's protection against unintended pregnancy and HIV*/STDs† were observed.
Molla *et al.*	2008	3743	Cross-sectional survey among Ethiopian youth	Those who did not believe in traditional values of preserving virginity (adjusted hazard ratio = 2.91), were more likely to have premarital sex than their counterparts

* HIV = Human Immunodeficiency Virus

† STD = Sexually Transmitted Diseases, OR = Odds Ratio

§ UNAIDS = United Nations Joint Program on HIV/AIDS

‖ AIDS = Acquired Immuno deficiency Syndrome, HAART = Highly active antiretroviral therapy

** WHO = World Health Organization

††AOR = Adjusted odds ratio

### Violence

Violence against women can occur in different settings including civil wars, communal disputes, and domestic violence.[4,5] The gender based violence (GBV) defined in this article includes all forms of physical, sexual, and emotional abuse against women. A cross-sectional survey in eight African countries revealed that 18% of the women had experienced GBV within one year.[6] Another multicenter study in ten countries (n-24,097) reported the lifetime risk of inter-partner violence (IPV) to be in the range of 15-71%, indicating a wide range of variation in communities.[7]

It is established that HIV is more likely to be transmitted in an act of forced penetration rather than during voluntary sex, due to factors such as inability to negotiate on safer sex and breach of epithelial barriers (with trauma) making it easier for the virus to enter the blood stream.[8] Forced sex increases the woman's chances of contracting a sexually transmitted disease (STD) four-fold.[8]

#### Violence makes women vulnerable to HIV

A population-based survey in Tanzania concluded that women had a significantly higher risk of HIV [Odds ratio(OR)-2.0] if they had experienced coerced sex before 18 years of age.[9] Fonck *et al.*[10] reports a two-fold higher risk of lifetime intimate partner violence for HIV positive women. A cross-sectional survey by Dunkle *et al.*[11] in South Africa has shown that intimate partner violence (OR-1.48) and high level of male control in a relationship (OR-1.52) was associated with HIV infection.

However, Jewkes *et al.*[12] has shown that while IPV is strongly associated with many HIV risk factors, it was not directly or significantly correlated with HIV positivity when corrected for other risk behaviors. A similar conclusion of an indirect relationship between IPV and HIV is suggested by Pettifor *et al.* and Langen *et al.*[13,14] This difference can be partly explained by the sample selection (general population vs. at-risk population). Many risk factors having a direct association with HIV, such as, age gap between the partners, frequency of intercourse, and number of sexual partners in the past year can be linked to IPV as well. In fact, Jewkes *et al.*[12] have demonstrated an association between such HIV risk factors and IPV, although a direct association has not been seen between IPV and HIV positivity.

An interventional prospective study in South Africa, by Pronyk *et al.*,[15] showed that economic empowerment and skill development of women reduced IPV by 55% in the intervention group. The microfinance initiative was linked with an HIV awareness program. However, neither the risk behaviors nor the HIV prevalence showed a significant difference at the end of follow up (after three years) between groups.

Studies outside Africa report a strong association between violence and HIV risk. In a cross-sectional survey of adolescents in USA, Viosin *et al.*[16] have reported that those exposed to family or community violence are three to four times more likely than their peers to engage in HIV risk behaviors. However, this association was more common in boys than in girls. In a survey of Hispanic women in Boston, USA, Raj *et al.*[17] have reported increased HIV risk for abused women than non-abused women. In India, a systematic multistage survey delineated a close relationship between IPV and risk behavior [men with extramarital affairs and STD symptoms were more likely to abuse their wives (OR-6.22 and 2.43, respectively)].[18]

In addition to IPV, women are victimized by other means, especially in times of war and civil unrest. Rape of women is well-documented in many civil wars in Africa (Rwanda, Sierra Leone, and Liberia). In some situations women were deliberately raped by HIV positive individuals to inflict psychological trauma and a ‘painful’ death.[5] In Nepal, at the beginning of the Maoist insurgency (1999), the HIV prevalence in Kathmandu stood at 2.7%. Three years later it had shot up to 17%.[19] Vulnerability of refugee women for transactional sex was identified as a risk factor for HIV in a survey of the Lugufu refugee camp (Tanzania) by Rowley *et al.*[20] A significant proportion of refugees reported forced sex (*P* < 0.001) compared to the villagers outside the camp. Many instances of forced sex had occurred after the displacement. Younger age of sexual initiation for boys, poverty and high-risk partners in the 15-24 age group were identified as risk factors for HIV among refugees (compared to villagers), in addition to vulnerability of women. Interplay of these risk factors may result in an increased rate of unprotected intercourse (by force or by consent) within the camps. In this sample, 20% of the refugees admitted to transactional sex (92% of instances since displacement) as opposed to 4% among villagers. However 82% of these encounters were with a fellow refugee rather than an outsider. Similarly, although the incidence of forced sex was high among refugees (10 vs. 4%), in many instances (64%) the perpetrator was the intimate sexual partner.

Sex trade is another form of female exploitation and HIV spread. Child sex and trafficking of women for prostitution is a burden for many countries. The Indo-Nepal border is notorious for such activity and it is estimated that 5000-7000 Nepali women are taken across the border to India annually in sex trafficking.[21] Subsequent analysis of the sero-status of such sex-trafficked Nepali women have shown disproportionately higher rates of HIV infection compared to native Indian sex workers.[22,23] While many studies and interventions have targeted female sex workers (FSWs), few have observed the STD profile and HIV risk of male clients of FSWs. They are an important source of spreading the infection via heterosexual intercourse. Recent studies in Mexico, China, and India have all shown high risk behaviors and fairly high rates of HIV and other STDs in this group.[24–26]

#### HIV positivity makes women vulnerable to violence

Violence following disclosure of the sero-status is not a universal phenomenon. It depends on the locality studied. Studies in United States have failed to demonstrate a higher rate of domestic violence against HIV-positive women when compared to sero-negative women.[27–29] In a study by Gielen *et al.*[30], only 4% of the sample reported physical abuse immediately following disclosure. Still 45% reported some form of abuse since the time of disclosure.

A study in Tanzania showed that HIV-positive women had a higher risk of physical and sexual violence than HIV-negative women.[31] Violence was more likely to occur when the sero-status of the partner was unknown. In a prospective study in Kenya, 10% of the women experienced violence or disruption of the relationship following disclosure.[32]

### Poverty

In a statistical analysis of UNAIDS data from 77 countries, Talbott *et al.*[33] have identified that income inequality within a country has a positive correlation with HIV prevalence. Several studies reporting a link between violence and HIV also report a correlation between poverty and HIV.[9,20,34] Limited financial power limits autonomy in sex for women. Langen *et al.*[14] reports that women who are economically dependent on their partners are less likely to insist on condoms during sex. There are several other studies conducted in Sub Saharan Africa that correlates low socioeconomic status with a high HIV risk.[35–37]

Food insufficiency is a marker of poverty that places women at a significant disadvantage to acquire HIV. In a population-based, cross-sectional study in Swaziland and Botswana, Weiser *et al.*[38] report that food insufficiency is associated with many risk behaviors for HIV (inconsistent condom use with a non-primary partner [Adjusted odds ratio (AOR) -1.73], intergenerational sexual relationships (AOR-1.46), sex exchange (AOR-1.84), and lack of control in a sexual relationship (AOR-1.68)). Interestingly, men reported fewer instances of food insufficiency and a lesser correlation between that and risk behaviors. Dunkle *et al.*[35] and Oyefara[39] have independently confirmed some of these associations with food insufficiency.

Several studies outside Africa, have demonstrated a positive correlation between HIV risk and poverty, for women. A study in China on FSWs has shown that some entered the trade voluntarily.[40] However, the driving force for selling sex was poverty. If social poverty was avoided it is assumed that these women would not sell sex and will not be at risk of acquiring HIV. Many ‘low end’ FSWs in India (streetwalkers) are unable to negotiate with the client to minimize risk behaviors. Unfortunately they account for a larger proportion of all FSWs.[41] Interestingly in Australia, a wealthier country, no difference in lifetime self-reported STDs have been observed in streetwalkers, private and brothel based sex workers.[42]

Migration for economic reasons is another risk factor for HIV in both sexes.[43] Women have become a sizable proportion of the migrant population, which may have a significance in the feminization of the epidemic.[44,45] Migrant male workers’ risk of acquiring an STD has been linked to the time spent in the host country.[46] In the South Asian context, these men are an important source of infection for women (both spousal and non-spousal partners).[46,47]

The studies by Oyefara[39], Dunkle *et al.*[35] and Weiser *et al.*[38] need special mention as they have not restricted the measures of poverty to family income. There may be differences in the way income is distributed in a family, which may be disadvantageous to the women. This phenomenon may create a state of ‘relative poverty’ for women and female children (depending on the gender norms of the society) although the family income is reasonable. Pronyck *et al.*[15] in their interventional study failed to show a reduction in HIV incidence or risk behaviors in the intervention group, despite the upliftment of economic standing, which may be a result of the above phenomena. The study in Botswana and Swaziland showed little correlation between low socioeconomic status and HIV risk, while there was a significant risk correlation for food insufficiency.[38] In fact food insufficiency may be a better marker of poverty as it represents a final common pathway of poverty than income. It is important to identify similar markers of poverty in different societies that might better correlate with HIV risk than wealth.

### Gender norms and culture

Gender norms can be defined as appropriate behaviors, beliefs, attitudes and conduct per gender as directed by society. It is a learnt behavior. In assigning gender roles, the phenotypical differences of genders are redefined as feminine and masculine with different capabilities in societal functioning (e.g., division of labor, power sharing, economic responsibility, dominance and submission).[48] Such gender norms are different in various societies and subject to change with time.[49] Yet, in many countries, especially in the developing world where HIV is spreading fast, females serve a subservient role to men as dictated by gender norms and culture.[50,51] These traditional norms place women at an increased risk as they have less freedom in choosing their partners, initiating and pacing sexual activity and negotiating on safer sex.[50,51] In addition, some traditions, customs and beliefs are also harmful to women (wife inheritance, having sex with a virgin as a cure for HIV).[52,53]

In our review, we have identified several studies linking gender roles to risk behavior. In a cross sectional analysis of females in detention in Georgia, USA, Voisin *et al.*[54] concludes that gender norms favoring male dominance as well as high-risk peer norms were significantly associated with STD risk (*P* < 0.05). A cross-sectional analysis by Harrison *et al.*[55] in South Africa gives an insight on changing gender roles and their impact on HIV. Their results were different to what was expected. Among men, hyper-romanticism and favoring of male dominance were associated with more consistent condom use (especially with the primary partner). More open sexual ideology was associated with less consistent condom use. Interestingly, more open sexual norms and hyper-romanticism in women was significantly associated with more partners. Similar unexpected findings were reported by Kaufman *et al.*[56] They showed that endorsement of traditional male dominant gender norms were associated with less HIV risk behavior for men. However, a negative attitude toward women showed (as expected) a positive correlation with risk behavior.

Qualitative studies may be a better tool to assess the complex interactions between gender norms and HIV, although the generalizability of findings remains doubtful. The interference of prevalent gender norms with HIV-prevention strategies is delineated by Go *et al.*[50] in a qualitative study in slum communities of Chennai, India. Risk behaviors such as multiple partners for men were ‘acceptable’ by the prevalent norms. Using condoms with regular partners was rare even when women were aware of its protection (as it would imply questioning a man's fidelity). Refusal of sex with husband was not an option even when the wife perceived a risk of infection. Similar findings were reported by Hebling *et al.*[57] Despite being educated on HIV transmission risks, women were unable to take precautionary measures due to prevalent gender norms and male dominance. In a study on African American adolescents in USA, Kerrigan *et al.*[58] explain how the female gender ideologies of care taking and emotional strength (standing by the man) can make them value relationship intimacy more than protection against HIV/STDs.

Despite their obvious negative impact on AIDS control, the traditional norms may have served a protective role against HIV too. In many developing nations, premarital sex is discouraged by tradition and is highly stigmatized. The ‘virginity’ of a female is still expected till marriage in many Asian and African societies.[49,59,60] Though it is a marker of discrimination against women, the fear of such discrimination may have discouraged premarital sex among adolescents helping to keep the epidemic at bay. It is interesting to note how the changing gender norms have made an impact on premarital sex, virginity norms, and HIV prevalence.

### Gender differences in adherence to prevention and treatment measures

It is a commonly held view that women have less access to HIV/STD prevention programs, especially in developing nations. In three high prevalence African countries (Burkina Faso, Ghana, Zambia), a better knowledge of HIV in females was associated with living in a community with more opportunities (better education, higher age in marriage and initiation of sex).[61] A positive association between HIV knowledge and education has been reported in several other studies in Africa.[37,62–65] A community-based study in rural Malawi has shown that while both sexes had a good knowledge of transmission of HIV/AIDS, men were more knowledgeable than women. Age, education, occupations other than farming, and childhood residency in a city were associated with better knowledge.[66]

The few studies conducted outside Africa (within our search limitations) showed conflicting results. A cross-sectional analysis among university students in Afghanistan showed that only 28.3% of students had a good knowledge of AIDS and the scores were better in females.[67] A similar study in China has shown that the overall knowledge scores were significantly high in males compared to females (*P* < 0.0001) and a better awareness was associated with the number of years spent in the university and coming from an urban area.[68] Yet, the level of education had a minimal impact in certain situations. A survey among secondary school girls in New Delhi, India has shown gross inadequacy of HIV- and STD-related knowledge (30% thought HIV can be cured, 21% thought oral contraceptive pills (OCP) would protect against HIV).[69]

The importance of educational interventions for women has been underscored in several studies.[37,70–73] Diclemente *et al.*[70] in a randomized controlled trial demonstrated that adolescent girls who underwent a comprehensive educational package on HIV/AIDS had significantly less risk behaviors in a 12-month follow-up period. Gavin *et al.*[37] in their study on adolescent females in Zimbabwe have shown that those who had sex education in schools had a decreased risk of HIV (OR-0.43). Chhabra *et al.*[72] and Hecej *et al.*[71] have reported better risk reduction practices in adolescent females compared to males following an educational program in India and Croatia, respectively. Interestingly a similar program targeting a different high-risk group in China (clients of FSWs) has shown promising results, with a significant increase in those using prevention services (OR-2.2).[24]

HIV testing, risk perception, and disclosure of sero-status are also reported to show a gender difference. Age, literacy, good doctor-patient relationship were associated with disclosure for men while having children, and being religious were associations for disclosure in women.[74] A cross-sectional survey among African American adolescents in the US has shown that females were significantly more likely to undergo voluntary HIV testing (OR-2.4 to 2.7) than males.[75]

However, several studies involving sexually active females have shown that a good knowledge of HIV is not synonymous with avoiding risk behavior.[62,63,66] In other words the knowledge is not put to practice. There are two main reasons for this paradox, impaired risk perception[62,63] and social barriers, that limits sexual autonomy for women. Some authors suggest a gender difference in risk perception as well.[66]

The paradoxical impact of education on HIV in the early 1990s in Africa is worth mentioning. HIV was associated with a better education in the early days of the epidemic in Africa. Some authors explain this phenomenon by the hypothesis that better educated had better jobs, more money, and more access to paid sex and IV drug use.[76] Education without an effective sex education component may have a minimal role in countering the epidemic. Asia today, may be in the state Africa was two decades ago.

### Highly active antiretroviral therapy

In this section we discuss the results of studies that have looked into a gendered difference in HAART-related issues (availability, accessibility, and adherence), excluding those related to the biological differences of sexes (side effects and clinical and viral response).

An analytical study of 23 sero-converter cohorts from Europe, Australia, and Canada have shown that women fared better after HAART was introduced. Comparing the pre-HAART (before 1997) period and the HAART era, authors have reported a lesser risk of AIDS and death in women compared to men (OR-0.76 and 0.68, respectively).[77] Moore *et al.* and Chen *et al.* have both reported a better outcome for females in two cohorts on HAART.[78–79]

It is speculated that women in resource-limited settings may have restricted access to treatment due to socioeconomic constraints and gender norms.[80] However, the evidence is contrary to this. Chandra *et al.*[81], Muula *et al.*,[82] and Braitstein *et al.*[83] have all shown that women are not at a disadvantage in access to HAART, in resource-limited settings. The epidemiology update of UNAIDS for 2008 confirm these findings. In 45 developing countries (with the exception of Chile and Belize) the coverage was better for females with HIV. Still, it is important to note that the overall coverage of HAART in these settings was low. In many countries, coverage of the HIV-positive population with HAART was less than 40% for both men and women. Only 35% of pregnant women had access to the therapy in 2008.[1]

Adherence to therapy is another dimension of interest. Several authors have reported women to be less adherent to HAART.[84–88] Several possible associations with this observation are depression,[84,85] alcohol dependence,[88,89] other substance misuse,[85,90] and increased side effects in women.[91]

## DISCUSSION

In summary, as expected, violence, gender norms, and poverty placed women at a disadvantage with regard to HIV/AIDS (increased risk of contracting HIV and increasing the burden of those living with the disease). However, regarding access to HAART, females fared better than males in many parts of the world.

In interpretation of the available evidence, the following issues need attention:

The vulnerability of sex-trafficked women, prostitutes, and victims of rape to HIV is obvious. However, the rise in the rate of infections at times of war and displacement is simply not a case of women being raped by the ‘enemy'. It may be the result of a complex interaction of several risk factors like, transactional sex, poverty, lack of opportunities, breakdown of civil order, and less rigid sexual norms.

Many studies only demonstrate that HIV positive women were significantly subjected to violence compared to the HIV-negative population. It is not clear whether the violence increased or began after sero-status disclosure. More studies are needed to identify which comes first; violence or HIV, or is it a vicious cycle.

Poverty and its relation to HIV is simply not an issue that can be reduced to the availability of money. Low income countries obviously may have many risk factors for HIV spread such as illiteracy, unemployment, and lack of opportunities for women. Yet, countries with a better income also have their own risk factors (fast life style, overcrowding in cities, and prostitution). Even in the affluent countries there may be microenvironments that are disadvantaged and not any different from the developing world with regard to HIV risk. When the data are generalized to the whole country, these significances go unnoticed. This argument is further supported by the findings of Talbott *et al.*[33] who showed that while the gross domestic product (GDP) of a country shows little or no relation to HIV/AIDS prevalence, the income inequality shows a positive correlation.

Regarding gender norms, there is the obvious need to discuss the conflicting or rather unexpected findings of some studies,[55,56] which do not collaborate with the view of high HIV transmission risk and traditional gender norms. This may be partly explained by the theory that traditional gender roles are changing in contemporary societies. Evidence for such change has been documented in the educated younger generations of South Africa.[92,93] Such contrasts may also be influenced by the improved awareness of the transmission of HIV. The more consistent use of condoms by dominant males may be an expression of responsibility or a measure for their own protection as they are likely to have multiple partners. More open sexual ideology being associated with less condom use may be an indicator of trust and selection of an educated and similar partner. Still the association of hyper-romanticism and open sexual ideology with more partners, in females, is difficult to explain. However some weaknesses in the study methodology may have influenced the outcome. The samples were not representative of the general population (Harrison *et al.*[55] used a sample with a disproportionate number of females in the ratio of 2:1, while Kaufman *et al.*[56] used a sample of high-risk males only). The sample sizes were limited to generalize the findings to a larger community.

On educational interventions and their gender implications, many studies have demonstrated a positive link between living in urban communities, education, and better HIV knowledge for women.[32,61–64] Faced with the epidemic, many Sub Saharan countries have initiated educational campaigns for the public, with funding from global agencies. The positive impact of such interventions are clearly visible, specially with regard to females.[76,94] However, the picture in the Asian context is less promising. Although many countries in Asia are still considered to be low prevalence, this can be a false state of complacency. Despite the fact that cultural barriers may prevent effective sex education at schools; the risk behaviors are high even among those having access to education.[67,69] Male dominance and gender norms limit a woman's ability to protect herself from infection despite being educated about HIV/AIDS. Therefore, we propose that a successful education program must concentrate on both sexes equally and be sensitive to prevalent gender norms in the society.

The evidence does not support the commonly held belief that women are at a disadvantage with access to HAART in resource-limited settings. This may be explained by the fact that women have two motives to enter a treatment plan to treat themselves and to prevent mother-to-child transmission.

There were several limitations in our review; it was limited to articles published in English only, and it is possible that important studies published in other languages were missed. Most of the data was from African and Asian countries; data from Latin American and Eastern European countries was minimal. Data on response to HAART was predominantly available from cohorts in developed countries. Such gaps in the available data affect the validity and generalizability of our conclusions.

We have recognized the following as areas with ‘gaps in knowledge’. These issues should be explored with further research.

1. Incidence and form of violence against HIV-positive women by intimate partner and others, following disclosure of sero-status in developing countries is not well explored.
2. Specific markers of poverty that may reflect the relationship between HIV and women better than wealth itself, needs to be investigated. Such markers may be different in each community (as food insufficiency was an indicator in some African countries).
3. Changing gender norms and their impact on women and HIV has been inadequately explored in Asia. The protective role (if any) of traditional gender norms and virginity norms needs to be identified.
4. Specific issues in different societies that cause discordance between knowledge and practice has to be analyzed as this has a direct impact in policy design on prevention.
5. Why do women have better access to HAART (as shown by statistics) despite the commonly held belief to the contrary? It is important to keep track of sex ratios on HAART as more people gain access to it, to avoid discrimination of women.
6. Identification of culturally appropriate educational tools to impart sex education for adolescents is a need of the hour.

## CONCLUSION

The gendered implications of many aspects of HIV are apparent. The traditionally held view that women are at a disadvantage is true with regard to several issues such as violence, poverty, and gender norms. In treatment-related issues, women (at least in Africa) have made significant gains with better awareness. The access to HAART was better for females in many resource-limited settings, although the overall coverage was unsatisfactory.

Much of the work done in the last decade has focused on the Sub Saharan Africa, which is the hub of infection with large numbers of patients. However, the social and cultural dynamics of Asia and Africa are quite different. Although many Asian countries (India, China) are considered to be low prevalence ones for HIV, the disease burden may actually be considerable in Asia for two reasons; the large population (even a smaller percentage equates to a large number of infections) and under-reporting. Given the potential reservoir of infection, the focus of research in the coming decade should be Asia.
